# Upper limb amputation due to a brachial arterial embolism associated with a superior mesenteric arterial embolism: a case report

**DOI:** 10.1186/1756-0500-5-372

**Published:** 2012-07-24

**Authors:** Tsuyoshi Yamada, Toshitaka Yoshii, Hideya Yoshimura, Koji Suzuki, Atsushi Okawa

**Affiliations:** 1 Section of Orthopedic and Spinal Surgery, Graduate School, Tokyo Medical and Dental University, 1-5-45 Yushima, Bunkyo-ku, Tokyo, 113-8519, Japan; 2 Global Center of Excellence (GCOE) Program for International Research Center for Molecular Science in Tooth and Bone Disease, Tokyo Medical and Dental University, 1-5-45 Yushima, Bunkyo-ku, Tokyo, 113-8519, Japan; 3 Section of Orthopedic Surgery, Kawaguchi Industrial General Hospital, 1-18-15 Aoki, Kawaguchi-shi, Saitama, 332-0031, Japan; 4 Section of Orthopedic Surgery, Toride Kyodo Hospital, 2-1-1, Hongou, Toride-shi, Ibaraki, 302-0022, Japan

**Keywords:** SMA, Embolism, Upper limb, Amputation

## Abstract

**Background:**

Acute mesenteric ischemia due to an embolism of the superior mesenteric artery is associated with a high mortality rate. Over 20 percent of acute mesenteric embolism cases consist of multiple emboli, and the long-term prognosis depends on the incidence of subsequent embolic events at other sites. The incidence of emboli in the upper extremity associated with a superior mesenteric arterial embolism has rarely been described. The signs and symptoms of ischemic change in the upper limb can be masked by other circumstances, such as postoperative conditions or complications. In these cases, a late presentation or delayed diagnosis and treatment can result in limb loss.

**Case presentation:**

We present a rare case of a 67-year-old Japanese woman with atrial fibrillation who developed an embolic occlusion of the brachial artery associated with a superior mesenteric arterial embolism. She developed gangrene in her right hand, which had progressed to the point that amputation was necessary by the time the gastrointestinal surgeon had consulted the Department of Orthopedic Surgery. The brachial arterial embolism diagnosis was delayed by the severe abdominal symptoms and shock conditions that followed the emergency enterectomy, resulting in amputation of the upper limb despite anticoagulation therapy. In this case, multiple infarctions of the spleen were also observed, indicating a shower embolism.

**Conclusions:**

When treating a superior mesenteric arterial embolism in a patient with atrial fibrillation, the possibility of recurrent or multiple arterial thromboembolic events should be considered, even after the procedure is completed.

## Background

Acute mesenteric ischemia due to an embolism of the superior mesenteric artery (SMA) is associated with a high mortality rate [1]. Over 20 percent of acute mesenteric embolism cases consist of multiple emboli, and the long-term prognosis depends on the incidence of subsequent embolic events at other sites. An embolic episode in a lower limb following an SMA embolism has been previously reported [2]. However, the incidence of emboli in the upper extremities associated with an SMA embolism has rarely been examined.

An embolus in an upper limb is an uncommon event; when diagnosed promptly, however, it can be successfully treated with prompt surgery, such as an embolectomy [3]. Therefore, early amputation of an arm due to an embolism is exceptionally rare [4]. It has been reported that embolisms accounted for approximately 1 percent of upper limb amputations [5]. The signs and symptoms of ischemic changes in the upper limbs can be masked by other circumstances, such as postoperative conditions or complications. In these cases, a late presentation or delayed diagnosis and treatment can result in limb loss.

We report a rare case of brachial arterial embolism associated with an SMA embolism. Because of the severe abdominal symptoms and the shock conditions that followed an emergency enterectomy, the brachial arterial embolism diagnosis was delayed, resulting in amputation of the upper limb.

## Case presentation

**Figure 1:**
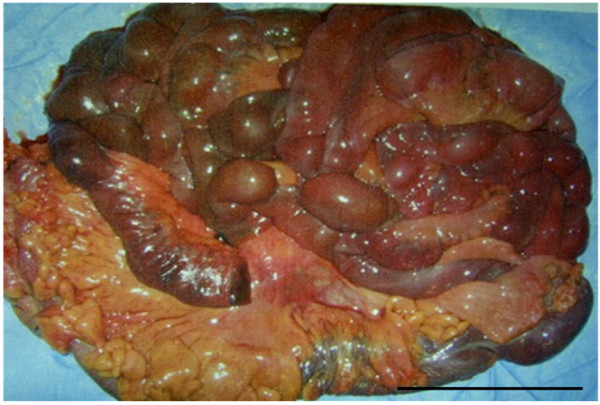
** Resection of the necrotic small intestines and right colon.** The small intestines and right colon were necrotic due to an embolic occlusion of the SMA. Black Bar: 10 cm.

A 67-year-old Japanese woman complained of acute-onset abdominal pain and vomiting (Glasgow Coma Scale score = 14). Her vital signs were largely stable, apart from tachycardia with atrial fibrillation (Af) that had never been treated. The laboratory data revealed acidosis, inflammation, and elevated lactate and creatine phosphokinase (CPK) levels. The contrast-enhanced computed tomography (CT) imaging revealed that the SMA was occluded by a thromboembolism. Because of her pre-existing Af, she was diagnosed with embolic occlusion of the SMA and intestinal necrosis. She was treated with an emergency enterectomy (Figure 1). The pulse of the SMA disappeared, and a relatively fresh thrombus was observed in the stump of the resected intestines. Anticoagulation therapy with heparin was initiated after the laparotomy.

Post-operatively, she developed methicillin-resistant *Staphylococcus aureus* (MRSA) colitis and sepsis. Due to her respiratory insufficiency and shock conditions, she was treated with ventilator support. Although antibiotics resolved her MRSA colitis within 3 weeks of the laparotomy, she began to gradually develop gangrene in her right hand.

**Figure 2:**
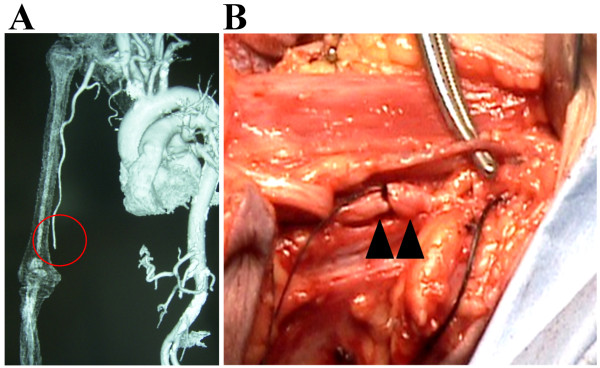
** Above-elbow amputation 6 weeks after the emergency laparotomy.** (**A**) The contrast-enhanced computed tomography. The brachial artery (circle) was occluded at the supracondylar level due to a thromboembolism. (**B**) An intraoperative photograph of the brachial artery. The arterial lumen is filled with an organized embolus.

The three-dimensional CT images showed that her right brachial artery was occluded 5 cm proximal to the elbow joint (Figure 2B); however, we could detect arterial pulsation 15 cm proximal to the elbow joint. We diagnosed her with an acute brachial arterial occlusion associated with an SMA embolism, and 6 weeks after the emergency laparotomy, an amputation was performed 15 cm proximal to the elbow joint. We did not observe any severe atherosclerotic changes in the brachial artery. The pulsation of the brachial artery disappeared at the level of the bone transection, and the arterial lumen was filled with an organized embolus (Figure 2B).

An abdominal CT scan revealed multiple infarctions of the spleen, indicating a shower embolism. Anticoagulation therapy was re-started after the amputation to prevent recurrent or new emboli. She was able to ambulate independently 2 months after the amputation. She used a prosthetic upper limb for 12 months after the amputation, at which time she died of malnutrition resulting from the enterectomy.

## Discussion

Acute mesenteric ischemia refers to the acute onset of intestinal hypoperfusion, which can be caused by occlusive or non-occlusive obstruction of arterial or venous blood flow. SMA emboli are a major cause (up to 50 percent) of acute mesenteric ischemia [6]. The SMA is anatomically susceptible to embolisms because of its large caliber and narrow take-off angle from the aorta. Acutely insufficient mesenteric arterial blood flow results in mortality rates that exceed 60 percent. Even after thrombolytic therapy or bowel resection, SMA mortality rates exceed 25 percent [2,7]. Over 20 percent of acute mesenteric emboli occur with other embolic events. SMA emboli are associated with subsequent embolic events at other sites, such as shower embolisms. Embolic episodes following an SMA embolism can occur in the heart, brain, or a lower limb [2]. As we described in this report, an upper extremity embolism can occur secondary to an SMA embolism, although this is a rare event.

The most frequent causes of upper limb amputation are trauma and cancer, followed by vascular complications. Amputating an arm following an embolism is rare because emboli are the cause of only 1 percent of upper limb amputations [5]. In most upper limb embolism cases, it is not difficult to detect the emboli [4]. Duplex imaging, Doppler ultrasonography, and angiography may be performed to confirm the clinical diagnosis and the suspected location of the emboli. Prompt (within 24 hours) operative intervention, such as an embolectomy with a Fogarty catheter, can produce satisfactory results. The embolectomy procedure successfully restores circulation in 65–94 percent of patients [8].

The signs of ischemic change in an upper limb can be masked by the patient’s post-operative condition or by complications, which can lead to a delayed diagnosis. The limb remains threatened once the ischemic cascade has been initiated, even after successful revascularization [9]. In the case presented here, a patient with co-morbid Af suffered an SMA embolism. The patient complained chiefly of abdominal pain and required post-operative mechanical ventilation. She developed gangrene in her right hand, which had progressed to the point that amputation was necessary. A brachial arterial embolism, SMA occlusion and multiple infarctions of the spleen were observed, indicating a shower embolism. As a result of the late presentation, late consultation and delayed embolectomy treatment, she underwent an above-elbow amputation despite anticoagulation therapy.

Upper limb emboli may be attributed to a variety of sources. Two-thirds of the cases are of cardiac origin, and Af is the usual etiology. Obstructions have been found in the subclavian artery in 7–8 percent of patients, at the axillary level in 26–36 percent of patients, at the brachial level in 48–52 percent of patients, and distal to the elbow in 9–15 percent of patients [4,10-13]. In our case, the obstruction was at the brachial artery level. We performed an amputation 15 cm proximal to the elbow joint. When the condyles cannot be preserved, an amputation may be performed in the distal third of the humerus. This amputation level allows fitting a variety of passive, body-powered, myoelectric, and activity-specific elbow prostheses and allows an adequate length to suspend and control the prostheses [14].

## Conclusions

When treating an SMA embolism in a patient with Af, the possibility of recurrent or multiple thromboembolic events should be considered, even after the procedure. Rapid diagnosis is essential for preventing the catastrophic events associated with intestinal infarction and the subsequent development of shower emboli.

## Consent

Written informed consent was obtained from the patient’s next of kin to publish this case report and any accompanying images. A copy of the written consent is available for review by the editors of this journal.

## Abbreviations

SMA, Superior mesenteric artery; Af, Atrial fibrillation; CPK, Creatine phosphokinase; CT, Computed tomography; MRSA, Methicillin-resistant Staphylococcus aureus.

## Competing interests

The authors declare that they have no competing interests.

## Authors' contributions

TY1 was the major contributor to studying the case and writing the manuscript. TY1, HY and KS were involved in the patient’s medical care. TY2 and KS were responsible for drafting the manuscript. TY2 edited and submitted the manuscripts. TY2 and AO critically revised the manuscript and provided the final approval of the version to be published. All of the authors read and approved the final manuscript.
